# “Multidisciplinary fast-track” care can significantly reduce risk of mortality among hip fracture patients at least 80 years old: a single-center retrospective study

**DOI:** 10.1186/s12877-024-05183-y

**Published:** 2024-07-10

**Authors:** Yinbo Zhong, Mingxia Liu, Zhenzhen Cheng, Yuanyuan Yao, Yang Yu, Ge Luo, Bin Zheng, Min Yan

**Affiliations:** 1 Department of Anesthesiology, Second Affiliated Hospital, Zhejiang University School of Medicine, No. 88 Jiefang Road, Hangzhou, Zhejiang China; 2 Department of Anesthesiology & Clinical Research Center for Anesthesia and Perioperative Medicine, Huzhou Central Hospital, The Affiliated Huzhou Hospital, Zhejiang University School of Medicine, The Fifth School of Clinical Medicine of Zhejiang Chinese Medical University, Affiliated Central Hospital Huzhou University, Huzhou, Zhejiang China; 3 Department of Anesthesiology, The First People’s Hospital of Weifang, Weifang, Shandong China; 4 Department of Surgery, University of Alberta, Edmonton, Canada

**Keywords:** Older people, Hip fracture, Multidisciplinary fast track, Mortality

## Abstract

**Background:**

“Multidisciplinary fast-track” (MFT) care can accelerate recovery and improve prognosis after surgery, but whether it is effective in older people after hip fracture surgery is unclear.

**Methods:**

We retrospectively compared one-year all-cause mortality between hip fracture patients at least 80 years old at our institution who underwent hip fracture surgery between January 2014 and December 2018 and who then received MFT or conventional care. Multivariable regression was used to assess the association between MFT care and mortality after adjustment for confounders.

**Results:**

The final analysis included 247 patients who received MFT care and 438 who received conventional orthopedic care. The MFT group showed significantly lower one-year mortality (8.9% vs. 14.4%, *P* = 0.037). Log-rank testing of Kaplan-Meier survival curves confirmed the survival advantage. However, the two groups did not differ significantly in rates of mortality during hospitalization or at 30 or 90 days after surgery. Regression analysis confirmed that MFT care was associated with lower risk of one-year mortality (hazard ratio [HR] 0.47, 95% confidence interval [CI] 0.281–0.788, *P* = 0.04), and the survival benefit was confirmed in subgroups of patients with anemia (HR 0.453, 95% CI 0.268–0.767, *P* = 0.003) and patients with American Society of Anesthesiologists grade III (HR 0.202, 95% CI 0.08–0.51, *P* = 0.001).

**Conclusions:**

MFT care can reduce one-year mortality among hip fracture patients at least 80 years old. This finding should be verified and extended in multi-center randomized controlled trials.

## Introduction

By 2050, the global incidence of hip fractures is expected to exceed 6 million per year [1], and such fractures are associated with high mortality and morbidity, especially among the older people [1–7]. Older people are at far greater risk than young people of suffering hip fractures after falls, slips or minor trauma, which reflects loss of bone mass and muscle strength around hip joints during aging [8, 9]. Numerous studies point to the importance of surgical intervention as early as possible after hip fracture in order to optimize prognosis, particularly for patients at least 80 years old [6–9].

Consensus guidelines [7, 10] have begun recommending multidisciplinary collaborative diagnosis and perioperative management of hip fracture patients in order to accelerate surgical intervention and improve prognosis [11–18]. The term “multidisciplinary fast-track” (MFT) is often used to refer to the close cooperation of various clinical departments to diagnose, treat and rehabilitate patients after medical interventions [7, 10, 19]. While MFT care has proven effective at improving prognosis for patients undergoing various types of surgery [12, 13, 15], studies have reported mixed results for its effects on mortality among older people after hip fracture surgery [13, 17, 20, 21]. One reason for the mixed results may be that those studies examined a wide age range of patients, from 60 years and older.

We examined here whether MFT care can improve mortality specifically among patients 80 years and older after hip fracture surgery. We also wished to identify risk factors for worse survival among such patients, regardless of whether they received MFT or conventional orthopedic care.

## Materials and methods

This single-center retrospective cohort study was approved by the Ethics Committee of the Second Affiliated Hospital of Zhejiang University School of Medicine (approval I2019001002), which waived the requirement for informed consent.

### Patients

We retrospective analyzed medical records for patients who underwent hip fracture surgery at our medical center between January 1, 2014 and December 31, 2018 and who were at least 80 years old at the time of surgery. We excluded patients who had pathological or repeat fractures, multiple injuries or fractures, or high-energy trauma such as from an automobile accident.

### Perioperative management

Patients who were enrolled before May 2017 received conventional orthopedic care, i.e. the procedures for diagnosis, treatment and rehabilitation routinely applied to older people with hip fracture at our institution. Patients were examined and admitted to the orthopedic ward after diagnosis of hip fracture, where they underwent additional preoperative testing. If their condition was complex, specialists were consulted. This preoperative examination and assessment might take several days, reflecting the large patient volume at our medical center. As a result, patients typically underwent surgery more than 5 days after admission.

Patients enrolled from May 2017 onwards received MFT care. After admission to the emergency room, patients were examined within 24 h by a multidisciplinary team drawn from at least three departments, which always included the Departments of Emergency Medicine, Orthopedic Trauma, and Anesthesiology. The team focused on stabilizing and optimizing the patient’s preoperative condition and, if appropriate, scheduling surgery within 72 h of admission. Surgeries were conducted in the first available operating room, after which patients received early nutritional support and rehabilitation.

### Data collection and outcomes

Clinical data were extracted from the Electronic Medical Record System and “Do care” Anesthesia Information System at our hospital. Preoperative data included demographics, laboratory tests, American Society of Anesthesiologists (ASA) grade, fracture type, comorbidities, and Charlson comorbidity index [22]. Intra-operative data included the type and duration of surgery, type of anesthesia and volume of blood loss. Postoperative data included survival up to 12 months, postoperative complications (new-onset pneumonia, heart failure, deep vein thrombosis, pulmonary embolism), admission to the intensive care unit, length of hospital stay and total treatment costs. Patients were followed up by telephone for at least one year after surgery, and they were considered lost to follow-up if they or their family members could not be reached by telephone.

The primary outcome in this study was all-cause mortality within one year of surgery. Secondary outcomes included all-cause mortality during hospitalization as well as at 30 and 90 days after surgery, postoperative complications, postoperative admission to the intensive care unit, length of hospital stay, and total treatment costs.

### **Statistical analysis**

We estimated the minimal size of the MFT and conventional groups to be 215 in order to detect a difference of 12% in one-year postoperative mortality with statistical power of 90% at an alpha level of 5%. The difference of 12% was based on a previous study of older hip fracture people who received MFT or usual care [23].

Continuous data in this study showed a skewed distribution, so they were reported as median (interquartile range), and differences between the two groups were assessed for significance using non-parametric testing. Categorical data were reported as n (%), and intergroup differences were assessed using the chi-squared or Fisher’s exact test.

One-year survival curves were generated using the Kaplan-Meier method and compared using the log-rank test. Regression analysis was performed to identify variables associated with one-year mortality: variables that were associated with *P* < 0.1 in univariate regression were then included in multivariate Cox regression to control confounding. Results were reported, where appropriate, as hazard ratios (HRs) or odds ratios (ORs) and associated 95% confidence intervals (CIs). Regression was repeated for subgroups of patients stratified by sex, anemia, and ASA grade.

All statistical analyses were performed using SPSS 25.0 (IBM, Chicago, IL, USA) or R 4.1.0 (https://www.r-project.org). Results associated with *P* < 0.05 were considered significant.

## Results

**Fig. 1:**
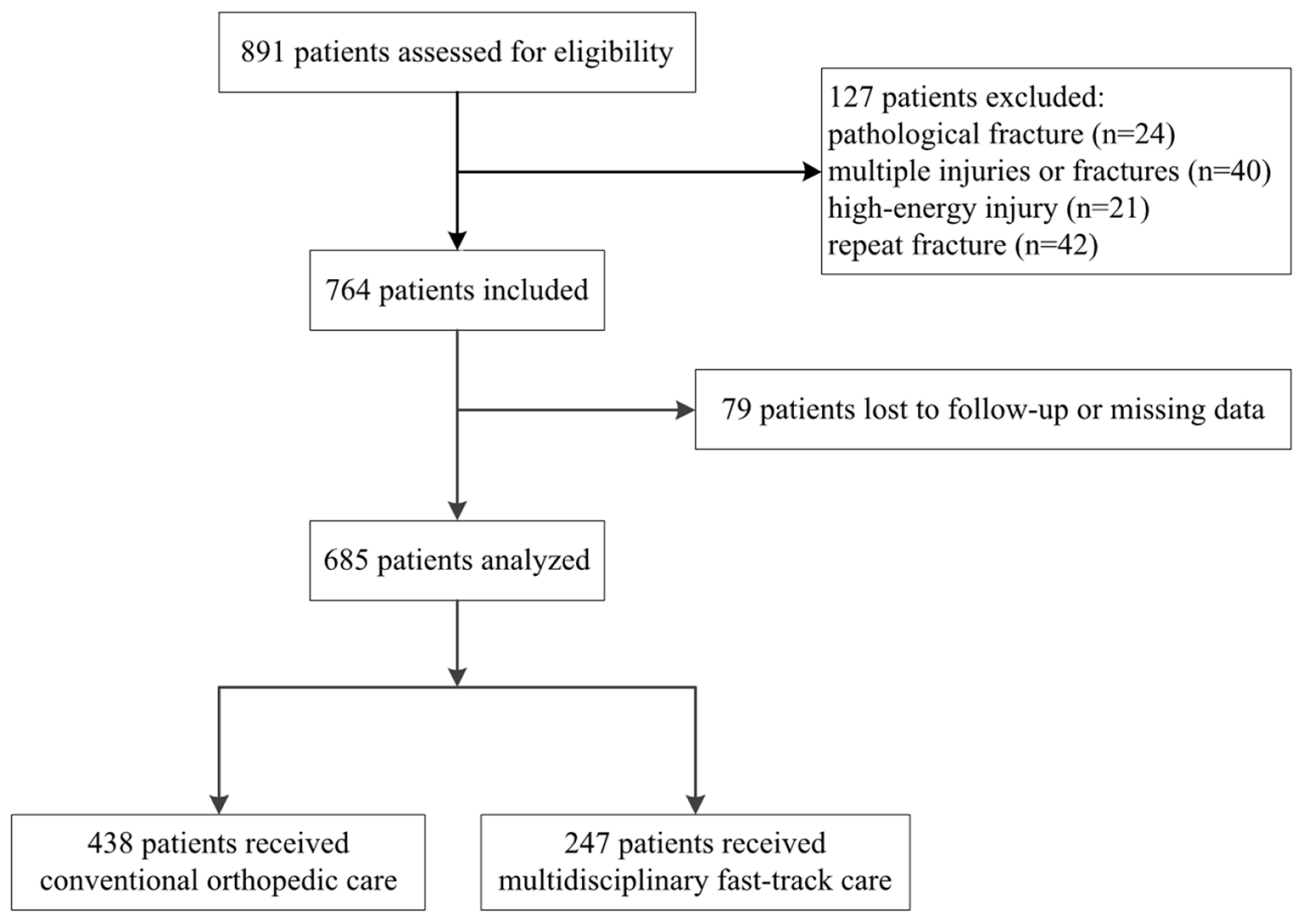
Flow diagram of patient selection and group allocation

Of the 891 patients who underwent hip fracture surgery at our medical center during the enrollment period, we included 685, comprising 247 in the MFT group and 438 in the conventional group (Fig. 1). The two groups did not differ significantly in age or sex, with women accounting for more than 74% of patients in each group (Table 1). The two groups did not differ significantly in most other clinicodemographic variables examined, except that the MFT group showed significantly higher prevalence of pneumonia (39.3% vs. 26.7%, *P* = 0.001) and chronic obstructive pulmonary disease (9.3% vs. 3.9%, *P* = 0.004). A significantly larger proportion of the MFT group received regional anesthesia.

**Table 1:** Clinicodemographic characteristics of patients

Characteristic	Conventional care (*n* = 438)	MFT care (*n* = 247)	*P*
Age (years)	85 (82–89)	86 (83–89)	0.095
Body mass index (kg/m^2^)	20.13 (17.78–21.80)	19.8 (17.58–22.87)	0.563
Sex			0.606
Male	104 (23.7)	63 (25.5)	
Female	334 (76.3)	184 (74.5)	
ASA grade			0.234
I-II	106 (24.2)	70 (28.3)	
III-IV	332 (75.8)	177 (71.7)	
**Preoperative laboratory tests**			
Hemoglobin (g/L)	104 (89–119)	108 (92–120)	0.177
White blood cells (10^9^/L)	7.4 (5.7–8.9)	7.4 (6.3–9.1)	0.18
Platelets (10^9^/L)	171 (133–222)	161 (106.6–161)	0.294
Albumin (g/L)	33.9 (31.1–36.1)	33.6 (31.3–35.9)	0.692
Creatine (µmol/L)	60 (49.5–77)	62 (51–79)	0.209
C-reactive protein (mg/L)	52 (27-78.25)	48.4 (25.4–65.9)	0.12
**Preoperative comorbidities**			
Pneumonia	117 (26.7)	97 (39.3)	0.001
Chronic obstructive pulmonary disease	17 (3.9)	23 (9.3)	0.004
Deep vein thrombosis or pulmonary embolism	9 (2.1)	5 (2.0)	0.978
Hypertension	238 (54.3)	143 (57.9)	0.368
Coronary heart disease	43 (9.8)	25 (10.1)	0.898
Heart failure	8 (1.8)	1 (0.4)	0.223
Atrial fibrillation	19 (4.3)	18 (7.3)	0.101
Stroke	57 [13]	27 (10.9)	0.425
Alzheimer’s disease	16 (3.7)	15 (6.1)	0.143
Parkinson’s disease	11 (2.5)	2 (2.4)	0.947
Diabetes mellitus	72 (16.4)	38 (15.4)	0.718
Malignant tumor	22 (5.0)	18 (7.3)	0.225
Renal insufficiency	16 (3.7)	10 (4.0)	0.795
Charlson comorbidity index			0.869
0	288 (65.8)	159 (64.9)	
1	103 (23.5)	58 (23.7)	
2	37 (8.4)	21 (8.6)	
3	8 (1.8)	5 (2.0)	
4	1 (0.2)	2 (0.8)	
5	1 (1.2)	0	
**Type of fracture**			0.336
Femoral neck	256 (58.4)	135 (54.7)	
Trochanteric	182 (41.6)	112 (45.3)	
**Surgical parameters**			
Type of anesthesia			< 0.001
General	311 (71)	143 (57.9)	
Regional	59 (13.5)	61 (24.7)	
Combined	68 (15.5)	43 (17.4)	
Type of surgery			0.321
Replacement	389 (88.8)	213 (86.2)	
Fixation	49 (11.2)	34 (13.8)	
Intraoperative blood loss (mL)	100 (50–200)	100 (50–200)	0.053
Duration of surgery (h)	2 (1.58–2.5)	2 (1.67–2.42)	0.764

Data are median (interquartile range) or n (%), unless otherwise noted

ASA, American Society of Anesthesiologists; MFT, multidisciplinary fast-track

**Fig. 2:**
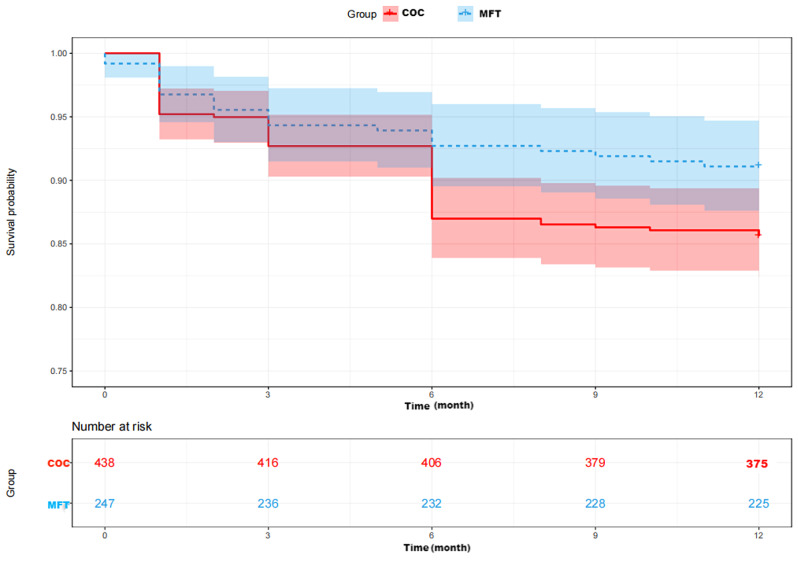
Kaplan-Meier curves of one-year survival among patients who received conventional orthopedic care or multidisciplinary fast-track (MFT) care. The shaded area indicates the 95% confidence interval around the corresponding curve

The MFT group showed significantly lower all-cause mortality at one year (8.9% vs. 14.4%, *P* = 0.037; Table 2), which was confirmed by Kaplan-Meier analysis (chi-squared = 4.12, *P* = 0.042; Fig. 2). However, the two groups did not differ significantly in rates of mortality during hospitalization or at 30 or 90 days after surgery.

**Table 2:** Comparison of primary and secondary outcomes between patients who received conventional or MFT care

Outcome	Conventional care (*n* = 438)	MFT care (*n* = 247)	*P*
All-cause mortality at			
one year	63 (14.4)	22 (8.9)	0.037
in hospital	7 (1.6)	3 (1.2)	0.944
30 days	21 (4.8)	8 (3.2)	0.332
90 days	33 (7.5)	14 (5.7)	0.354
Postoperative admission to intensive care unit	47 (10.7)	12 (4.9)	0.009
Postoperative complications			
Pneumonia	32 (7.3%)	18 (7.3%)	0.993
Heart failure	7 (1.6%)	3 (1.2%)	0.944
Deep vein thrombosis or pulmonary embolism	7 (1.6%)	2 (0.8%)	0.603
Total treatment costs (USD)*	6077.6 (5093.6-7374.6)	5745.5 (4584.9-6673.2)	< 0.001
Length of hospital stay (d)	10.83 (8.82–13.82)	8.89 (7.61–11.57)	< 0.001

Data are median (interquartile range) or n (%), unless otherwise noted

MFT, multidisciplinary fast-track

*Converted from Chinese RMB using the rate 1 RMB = 0.1437 USD

The MFT group showed a significantly lower rate of admission to the intensive care unit after surgery (4.9% vs. 10.7%, *P* = 0.009) and shorter intervals until surgery and discharge, leading to lower total treatment costs (Table 2). The two groups did not differ significantly in incidence of new-onset pneumonia, heart failure, deep vein thrombosis or pulmonary embolism.

Univariate regression identified 17 clinicodemographic variables that showed a *P* < 0.1 association with one-year all-cause mortality, of which 15 also emerged as significant in bivariate analysis (Table 3): age, sex, body mass index, ASA grade, hemoglobin level, albumin level, creatinine level, preoperative comorbidities, Charlson comorbidity index, fracture type, type and duration of surgery, type of anesthesia, intraoperative blood loss and MFT care. Based on the literature [7, 16, 22], we included the following 10 variables in multivariate logistic regression (Table 4): age, sex, pneumonia, hemoglobin level, albumin level, ASA grade, type of anesthesia, duration of surgery, Charlson comorbidity index and MFT care. Regression analysis linked MFT to significantly lower risk of one-year all-cause death after controlling for the other variables in the model (HR 0.47, 95% CI 0.281–0.788, *P* = 0.004). Across all patients in the study, risk of one-year all-cause mortality was significantly lower among women than men (HR 0.623, 95% CI 0.393–0.987, *P* = 0.044), higher among those with ASA grades III-IV than those with grades I-II (HR 6.238, 95% CI 3.649–10.664, *P* < 0.001), and lower among those with normal hemoglobin levels than among those with anemia (HR 0.985, 95% CI 0.973–0.997, *P* = 0.014), after controlling for the other variables in the model (Table 5).

**Table 3:** Comparison of clinicodemographic factors between study participants who died or not within one year after surgery, and bivariate regression to identify factors associated with mortality

Factor	Patient outcome		*P*	Bivariate regression	
	Survival (*n* = 600)	Death (*n* = 85)		HR	95% CI
Age (years)	85 (83–89)	85 (83–91)	0.02	1.055	1.008–1.103
Sex			0.01	0.557	0.357–0.869
Female	463 (77.2)	30 (35.3)			
Male	137 (22.8)	55 (64.7)			
Body mass index (kg/m^2^)	20.13 (17.8–22.2)	19.79 (13.5–29.6)	0.201	0.961	0.903–1.022
ASA grade			< 0.001	6.558	4.412–10.384
II	167 (27.8)	9 (10.6)			
III	426 (71.0)	54 (63.5)			
IV	7 (1.2)	4 (25.9)			
Hemoglobin (g/L)	107 (92–120)	96 (80–111)	< 0.001	0.975	0.964–0.987
Albumin (g/L)	33.9 (31.4–36.2)	31.8 (29.1–34.7)	< 0.001	0.886	0.840–0.935
Creatine (µmol/L)	61 (50–78)	64 (53-90.5)	0.008	1.006	1.002–1.010
Pneumonia			0.028	1.621	1.053–2.498
No	421 (70.2)	50 (58.8)			
Yes	179 (29.8)	35 (41.2)			
Heart failure			0.043	3.288	1.038–10.409
No	594 (99)	82 (96.5)			
Yes	6 [1]	3 (3.5)			
Deep vein thrombosis or pulmonary embolism			0.26	1.939	0.613–6.137
No	589 (98.2)	82 (96.5)			
Yes	11 (1.8)	3 (3.5)			
Malignant tumor			0.28	1.532	0.707–3.319
No	567 (94.5)	78 (91.8)			
Yes	33 (5.5)	7 (8.2)			
Charlson comorbidity index			0.133	1.209	0.944–1.549
0	395 (65.8)	52 (61.2)			
1	139 (23.2)	22 (25.9)			
2	51 (8.5)	7 (8.2)			
3	11 (1.8)	2 (2.4)			
4	2 (0.3)	1 (1.2)			
5	0 (0)	1 (1.2)			
Type of fracture			0.259	0.783	0.511-0.1.198
Femoral neck	347 (57.8)	44 (51.8)			
Trochanteric	253 (42.2)	41 (48.2)			
Type of surgery			0.078	0.444	0.180–1.096
Replacement	522 (87)	80 (94.1)			
Fixation	78 [12]	5 (5.9)			
Type of anesthesia			0.56	1.084	0.826–1.423
General	401 (66.8)	53 (62.4)			
Regional	102 [16]	18 (21.2)			
Combined	97 (16.2)	14 (16.5)			
Duration of surgery (h)	1.9 (1.6–2.4)	2.3 (1.8–2.7)	0.005	1.553	1.145–2.104
Intraoperative blood loss (mL)	100 (50–200)	100(50–200)	0.814	1	0.998–1.002
Perioperative care			0.048	0.612	0.377–0.995
Conventional	375 (62.5)	63 (74.2)			
MFT	225 (37.5)	22 (25.9)			

Data are n (%) or median (interquartile range), unless otherwise noted

ASA, American Society of Anesthesiologists; CI, confidence interval; HR, hazard ratio; MFT, multidisciplinary fast-track

**Table 4:** Multivariate Cox regression to identify factors independently associated with one-year all-cause mortality

Factor	β value	Standard error	Wald chi-squared	*P*	HR	95% CI
Perioperative care						
Conventional	Reference					
MFT	-0.754	0.263	8.216	0.004	0.47	0.281–0.788
Age	0.034	0.024	2.034	0.154	1.035	0.987–1.085
Sex						
Male	Reference					
Female	-0.474	0.235	4.059	0.044	0.623	0.393–0.987
ASA grade	1.831	0.274	44.788	< 0.001	6.238	3.649–10.664
Hemoglobin	-0.015	0.006	6.044	0.014	0.985	0.973–0.997
Albumin	-0.041	0.03	1.841	0.175	0.96	0.905–1.018
Pneumonia	0.135	0.231	0.34	0.56	1.144	0.728–1.798
Charlson comorbidity index	0.116	0.134	0.753	0.385	1.123	0.864–1.461
Type of anesthesia	0.08	0.142	0.313	0.576	1.083	0.819–1.431
Duration of surgery	0.195	0.165	1.404	0.236	1.216	0.880–1.679

ASA, American Society of Anesthesiologists; CI, confidence interval; HR, hazard ratio; MFT, multidisciplinary fast-track

**Table 5:** Bivariate and multivariate analysis to verify associations of MFT care with one-year all-cause mortality in different patient subgroups

Stratifying variable	Bivariate analysis			Multivariate analysis		
	Unadjusted HR	95% CI	*P*	Adjusted HR	95% CI	*P*
Sex						
Male	0.612	0.377–0.995	0.048	0.483	0.291–0.801	0.005
Female	0.44	0.227–0.852	0.015	0.409	0.208–0.803	0.009
Anemia						
Yes	0.651	0.394–1.073	0.092	0.453	0.268–0.767	0.003
No	0.284	0.034–3.361	0.244	0.371	0.040–3.430	0.382
American Society of Anesthesiologists grade						
II	0.021	0-5.199	0.17	0	-	0.961
III	0.204	0.081–0.512	0.001	0.202	0.080–0.510	0.001
IV	1.407	0.519–3.819	0.502	1.545	0.433–5.504	0.503

CI, confidence interval; HR, hazard ratio

After adjusting for other factors, MFT care was associated with significantly lower risk of one-year all-cause mortality in subgroups of patients with anemia (HR 0.453, 95% CI 0.268–0.767, *P* = 0.003) or ASA grade III (HR 0.202, 95% CI 0.08–0.51, *P* = 0.001).

## Discussion

Our single-center study suggests that MFT perioperative management of hip fracture patients at least 80 years old can significantly reduce risk of postoperative admission to the intensive care unit, shorten hospital stay, reduce medical costs and reduce risk of one-year all-cause mortality. We observed trends toward lower rates of mortality during hospitalization as well as at 30 and 90 days after surgery with MFT care, but these trends did not achieve statistical significance.

Our results support previous studies that found MFT care to shorten hospitalization and accelerate recovery among geriatric patients covering a wide age range from 60 years and older who underwent.hip fracture surgery [11–18]. At least in the present study, the advantages of MFT care are likely due in part to clinicians’ efforts to perform surgery within 72 h after admission. Delaying hip fracture surgery, especially among older people, may increase risk of postoperative mortality [24].

Previous work concurs with our finding of no clear benefit of MFT care on short-term survival [11, 14, 15, 25]. This may reflect that the primary risk factors for mortality soon after hip fracture surgery are age, sex, and preoperative complications [9, 26]. A meta-analysis concluded that treatment pathway was not significantly associated with short-term mortality of older people with hip fracture [27].

We found that our male older people, regardless of whether they received MFT or conventional care, were at higher risk of one-year mortality than female patients, which is consistent with previous studies [28–30]. This may reflect the greater prevalence of pneumonia, chronic obstructive pulmonary disease, smoking, and drinking among men [30].

We found that patients with anemia were at greater risk of one-year mortality than those with normal hemoglobin levels, regardless of whether they received MFT or conventional care. Consistently, previous work showed that normalizing low hemoglobin levels preoperatively can reduce the risk of one-year all-cause mortality in younger and older hip fracture patients [25, 26]. The present study and previous work highlight the need to optimize perioperative management of hemoglobin and nutrition for older people, especially those with known risk factors of anemia, such as metabolic disorders, blood loss, and chronic comorbidities [27].

We found that patients with ASA grades III-IV were at greater risk of one-year mortality than those with milder ASA grades, regardless of whether they received MFT or conventional care. This finding concurs with previous work identifying ASA grade as a predictor of postoperative mortality in older people with hip fractures [23, 31–33]. Our observation of ASA grade III as a “threshold” in risk of one-year mortality should be interpreted with caution, given that 70% of our study participants belonged to that grade. If this threshold can be verified in further studies, it may mean that patients with severe systemic comorbidities (ASA > III) are less likely than other patients to benefit from MFT care. This possibility should be explored in future work.

The present study focused on patients at least 80 years old, and its findings echo previous work on the benefits of MFT care for hip fracture patients at least 65 years old. In contrast to younger patients, our patients at least 80 years old were more likely to have life-threatening comorbidities or to suffer postoperative complications. One study has suggested that the risk of postoperative death among older people with hip fracture increases by 6% with every year of aging [23]. The similarities between patients below and above 80 years is interesting given that susceptibility to traumatic stress, time needed to recover from surgery, and overall risk of mortality increase strongly with age [34], while organ function and resilience decline [18, 35–37].

Our findings should be interpreted with caution given several limitations. First, the retrospective nature of the study prevented us from assessing the potential influence of events before admission or after discharge on postoperative mortality. In addition, nearly 90% of study participants had a Charlson cormobidity index of 0 or 1, so our results may not be generalizable to frailer patient populations. These low indices may underestimate actual comorbidity in our sample because certain conditions may not have been recorded appropriately. Third, we considered MFT care as a “whole” in our study without attempting to identify the influence of specific measures or practices on mortality. Given the lack of standardization about MFT care around the world, future work should examine particular MFT components that may be more effective at improving prognosis. Our results should be verified in larger, multi-center studies.

## Conclusions

MFT perioperative care can improve survival among hip fracture patients at least 80 years old. If these findings can be verified in larger studies, they argue for continuing research into MFT protocols for older people undergoing hip fracture surgery and for expanding such research to other types of orthopedic surgery.

## Data Availability

The datasets generated during and analyzed during the current study are available from the corresponding author on reasonable request.

## Abbreviations

ASA: American Society of Anesthesiologists
CCI: Charlson comorbidity index
CI: Confidence interval
COC: Conventional orthopedic care
HR: Hazard ratio
MFT: Multidisciplinary fast-track
